# A Cross-Sectional Study Assessing the Functional Status in Children With Juvenile Idiopathic Arthritis and Its Correlation With Their Quality of Life and Burden on Caregivers

**DOI:** 10.7759/cureus.66178

**Published:** 2024-08-05

**Authors:** Sangeeth Attuparambath, Srikumar Venkataraman, Asem R Chanu, Gita Handa, Narendra Bagri

**Affiliations:** 1 Physical Medicine and Rehabilitation, All India Institute of Medical Sciences, New Delhi, New Delhi, IND; 2 Pediatrics, All India Institute of Medical Sciences, New Delhi, New Delhi, IND

**Keywords:** family burden interview schedule, childhood health assessment questionnaire, caregiver burden, quality of life, functional status, juvenile idiopathic arthritis

## Abstract

Background

Juvenile idiopathic arthritis (JIA) is a common rheumatic disease in children, significantly impacting their functional status and quality of life (QoL), as well as imposing a burden on caregivers. This study aims to assess the functional status of children with JIA, their QoL, and the associated caregiver burden while exploring the correlations between these factors.

Methodology

A prospective, cross-sectional, observational study was conducted over 18 months. A total of 33 children diagnosed with JIA were evaluated using the Childhood Health Assessment Questionnaire (CHAQ), and Euro Quality of Life-5 Dimension-Youth (EQ-5D-Y). Caregiver burden was assessed using the Family Burden Interview Schedule (FBIS). Data were analyzed using descriptive statistics, regression analysis, and Spearman’s rank correlation.

Results

A total of 33 consecutive children with JIA were prospectively enrolled. The mean age was 10.1 ± 3.7 years, with a male predominance (63.6%, n = 21). Enthesitis-related arthritis was the most common subtype (42%, n = 14). The CHAQ scores indicated moderate disability, with profound impacts on walking and arising. Most children reported “some problems” in all EQ-5D-Y domains, with a mean health status visual analog scale score of 60.97 ± 23.43. The mean FBIS score was 9.64 ± 5.78, indicating a moderate caregiver burden. The majority of caregivers reported moderate financial, family routine, and family leisure disruptions. Significant correlations were found between CHAQ and EQ-5D-Y scores in several domains (p ≤ 0.040), as well as between specific CHAQ domains and FBIS scores (p ≤ 0.037).

Conclusions

Children with JIA experience significant functional limitations and reduced QoL, which also impacts their caregivers. Early rehabilitation and comprehensive care strategies are crucial for improving functional outcomes and QoL, as well as alleviating caregiver burden.

## Introduction

Juvenile idiopathic arthritis (JIA) is the most prevalent rheumatic disease in children, significantly affecting their quality of life (QoL) and imposing a substantial burden on caregivers [1]. It imposes significant challenges, causing pain, sleep disturbances, reduced quality of life, school absenteeism, and dependency on others for daily activities. Delayed and ineffective treatments can lead to joint deformities, exacerbating these issues and placing a substantial burden on families, particularly primary caregivers. Understanding the functional limitations, quality of life, and caregiver burden associated with JIA can facilitate better treatment planning, emphasizing early and effective therapy to enhance rehabilitation compliance.

The functional impairment and QoL of children with JIA are significant concerns. Functional status can be measured using tools such as the Childhood Health Assessment Questionnaire (CHAQ), which assesses the ability to perform daily activities [2]. The QoL in children with JIA can be assessed using instruments such as the Euro Quality of Life-5 Dimension-Youth (EQ-5D-Y) [3,4]. Furthermore, the impact of JIA on caregivers is profound, affecting their social, emotional, and financial well-being. Tools such as the Family Burden Interview Schedule (FBIS) can help quantify this burden [5].

The primary objective of the study was to assess the functional disability in children with JIA. The secondary objectives included assessing their QoL and caregiver burden and exploring the potential correlations of functional status with QoL and caregiver burden. Understanding these relationships may be crucial for developing comprehensive care strategies that address not only the medical but also the psychosocial aspects of JIA.

## Materials and methods

This prospective, cross-sectional, observational study was conducted in a single institutional setting from September 2020 to March 2022 after obtaining approval from the Institute Ethics Committee (approval number: IECPG-371/26.08.2020, RT-31/23.09.2020). The study included children diagnosed with JIA who visited the outpatient clinic during the study period. The inclusion criteria were children of either gender, under 18 years of age, symptomatic for more than six months (excluding systemic JIA), and caregivers able to read and comprehend Hindi or English. The exclusion criteria were unwillingness to participate, children receiving biological therapy, children or caregivers with intellectual disabilities, and children with other chronic systemic diseases or congenital/traumatic deformities.

Sample size

The sample size was calculated based on a study by Narayan and Muthuraja [6], with an expected proportion of children having disability set at 0.75, a relative precision of 15%, and a confidence level of 95%, resulting in a required sample size of 33.

Data collection

Written informed consent was obtained from the parents or guardians, and assent was obtained from children over seven years old. Clinical history, demographic data, and general physical examination of the subjects were recorded. Functional status was evaluated using the CHAQ, which measures difficulty in performing daily activities across the following eight domains: dressing and grooming, arising, eating, walking, hygiene, reach, grip, and activities [2]. The EQ-5D-Y was used to assess the child’s QoL across the following five dimensions: mobility, self-care, daily activities, pain or discomfort, and anxiety or depression [3,4]. The FBIS was employed to measure caregiver burden across various domains [5].

Statistical analysis

Descriptive statistics were used to summarize demographics and clinical characteristics. Functional disability was presented as proportions. Correlations between functional disability, QoL, and caregiver burden were analyzed using regression analysis and Spearman’s rank correlation test, with a two-tailed p-value <0.05 considered significant. Data were analyzed using SPSS Statistics Version 26 (IBM Corp., Armonk, NY, USA) software.

## Results

Demographics and clinical characteristics

A total of 33 consecutive children with JIA who fulfilled the inclusion criteria were enrolled using the convenience sampling method. The mean age was 10.1 ± 3.7 years, with a mean disease duration of 2.6 ± 1.5 years. There was a male predominance (63.6%, n = 21). The most common JIA subtype was enthesitis-related arthritis (ERA) (42%, n = 14), followed by polyarticular rheumatoid factor-positive arthritis (30%, n = 10) (Table 1). A total of 122 joints were affected in these children, with the knee joint being the most frequently involved (23%, n = 28), followed by the wrist joint (18%, n = 22).

**Table 1:** Distribution of subtypes of juvenile idiopathic arthritis stratified by age and sex. JIA: juvenile idiopathic arthritis; ERA: enthesitis-related arthritis; pJIA RF+: polyarticular JIA rheumatoid factor positive; pJIA RF-: polyarticular JIA rheumatoid factor negative; oJIA: oligoarticular JIA

Subtype	N	Female	Male	Male:female ratio	Mean age (years)
ERA	14	0	14	-	11.2 ± 3.8
pJIA RF+	10	8	2	0.25:1	9.5 ± 3.7
pJIA RF-	5	3	2	0.66:1	8.8 ± 3.5
oJIA Extended	1	0	1	-	15
oJIA Persistent	3	1	2	2:1	7.6 ± 3.0

Functional status, quality of life, and caregiver burden

The mean CHAQ score was 0.97 ± 0.53 (median = 0.88; range = 0-2.50), indicating moderate disability. The most affected domains were walking and arising, corresponding with the high involvement of the knee and ankle joints. The CHAQ scores measuring difficulty in performing daily activities across eight domains are highlighted in Table 2.

**Table 2:** CHAQ scores assessing functional status across eight domains. CHAQ: Child Health Assessment Questionnaire; SD: standard deviation

Domain	Mean ± SD	Median (range)
Dressing and grooming	0.70 ± 0.77	1.00 (0–3)
Arising	0.97 ± 0.88	1.00 (0–3)
Eating	0.73 ± 0.84	1.00 (0–3)
Walking	1.18 ± 1.01	1.00 (0–3)
Hygiene	1.00 ± 0.87	1.00 (0–3)
Reach	0.97 ± 0.77	1.00 (0–3)
Grip	0.76 ± 0.94	1.00 (0–3)
Activities	1.42 ± 1.06	1.00 (0–3)

The QoL assessment with the EQ-5D-Y instrument indicated that most children reported “some problems” across all domains. The frequency of responses in each domain of EQ-5D-Y is displayed in Table 3. The mean visual analog scale score for health status was 60.97 ± 23.43 (median = 62.00; range = 25-90).

**Table 3:** EQ-5D-Y scores across five domains. EQ-5D-Y: Euro Quality of Life-5 Dimension-Youth

Domain	No problems (level 1)	Some problems (level 2)	A lot of problems (level 3)
Mobility	12	13	8
Looking after myself	11	20	2
Doing usual activities	5	23	5
Having pain or discomfort	3	23	7
Feeling worried, sad, or unhappy	11	12	10

**Figure 1:**
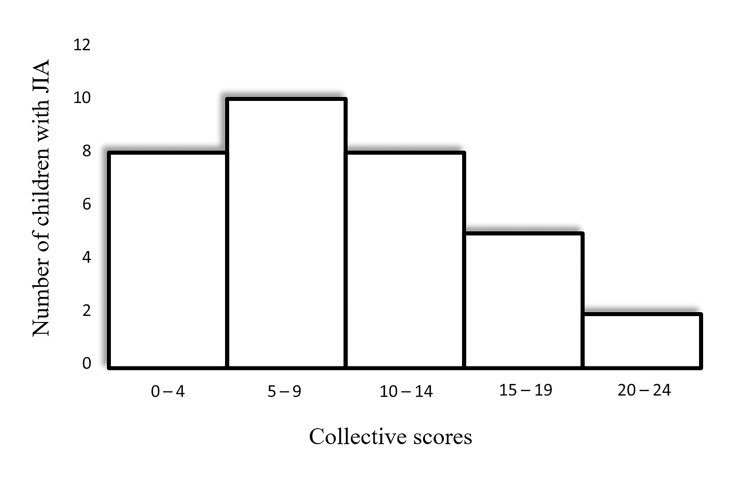
Frequency of collective scores for FBIS responses given by the caregivers. FBIS: Family Burden Interview Schedule; JIA: juvenile idiopathic arthritis

The mean FBIS score was 9.64 ± 5.78 (median = 9.44; range = 0-24.00), indicating a moderate burden to caregivers. The majority of caregivers reported moderate financial, family routine, and family leisure disruptions. The frequency of collective scores for FBIS responses given by the caregivers is shown in Figure 1.

Correlation analysis

Significant correlations were found between functional status (CHAQ scores) and QoL (EQ-5D-Y levels) in several domains, indicating that poorer functionality is associated with lower QoL. There were significant correlations between the CHAQ scores and Level 1 (p = 0.040) in the mobility domain, Level 1 (p = 0.023) and Level 2 (p = 0.007) in the looking after myself domain, Level 1 (p = 0.034) and Level 2 (p = 0.013) in the having pain or discomfort domain of EQ-5D-Y. Overall, 52% of the outcome in the CHAQ score could be defined by EQ-5D-Y values. There were also statistically significant moderate positive correlations between specific domains of CHAQ and FBIS scores, suggesting that certain functional limitations in children contribute to caregiver burden (Table 4).

**Table 4:** Significant correlations between functional status and caregiver burden. CHAQ: Child Health Assessment Questionnaire; FBIS: Family Burden Interview Schedule

CHAQ domain	FBIS questions	ρ	P-value
Dressing	The patient not helping with household work	0.452	0.008
Dressing	Stopping normal recreational activities	0.365	0.037
Eating	Neglect of the rest of the family due to the patient’s illness	0.404	0.020
Eating	Do other members get into arguments over this? How are they affected?	0.403	0.020
Grip	Any ill effect on the general atmosphere in the house?	0.435	0.012

## Discussion

This study aimed to understand the functional limitations and QoL in children with JIA and caregiver burden and explore the correlations between these factors. In our study, boys were found to be more commonly affected than girls, consistent with previous studies conducted in India [7-10]. This contrasts with studies from Western countries, where females were more commonly affected [11]. ERA was the most common subtype in this study, consistent with other Indian studies [7,9,10]. These findings contrasted sharply with those from Western countries, where the oligoarticular subtype has been reported with much greater frequency [12]. This finding may be attributed to several factors, including ethnic differences, delays in timely referral of JIA cases to tertiary care centers, selective exclusion of systemic-onset JIA from the study population, and the relatively small sample size of the study.

Functional limitations were prevalent among the children, with moderate disability indicated by CHAQ scores. This is similar to findings from the studies by Amine et al. and Abdelaleem et al. [13,14], but slightly higher than those reported by Tarakçı et al. [11]. The most affected CHAQ domains were walking and arising, correlating with the high involvement of the knee and ankle joints. This underscores the importance of targeted rehabilitation interventions to improve mobility and reduce disability.

The EQ-5D-Y results indicated that most children experienced “some problems” in all domains, reflecting a moderate impact on their QoL. This aligns with findings from the studies by Mańczak et al. and Nandi et al., where children with JIA had lower health-related QoL compared to healthy peers [15,16]. The significant correlation between CHAQ scores and EQ-5D-Y levels highlights the interdependence of functional status and QoL, suggesting that improving functionality could enhance overall well-being.

Caregiver burden was moderate, with significant correlations between specific functional limitations in children and caregiver burden in financial, family routine, and family leisure domains. This finding aligns with the studies by Gowda et al. and Khatun et al. which reported substantial emotional, social, economic, and labor impacts on caregivers [10,17]. In a study to assess psychological stress in caregivers of JIA children, Iwamoto et al. documented, using a different scale, that strain caused by environmental barriers accounted for the highest levels of stress among caregivers [18]. The domain of environmental barriers was not present in the FBIS instrument utilized in the current study. Nevertheless, the use of FBIS in the current study provided valuable insights into the specific areas where caregiver burden is most pronounced.

Despite the commendable results of the study, it is not free from limitations. There was a lack of randomization, and the cross-sectional design precluded assessment of changes over time. Additionally, the exclusion of children receiving biological therapy and those with systemic onset JIA may have limited the generalizability of the findings.

## Conclusions

Children with JIA experience significant functional limitations and reduced QoL, which, in turn, impacts their caregivers. Early and comprehensive rehabilitation interventions are essential to improve functional outcomes and enhance the overall well-being of both children and their families. A multidisciplinary approach involving medical, psychological, and social support will be crucial in enhancing the well-being of these children and their families.
